# Supplementary material for: Association between sociodemographic factors, clinic characteristics and mental health screening rates in primary care
Source: PLoS One. 2024 Mar 28;19(3):e0301125. doi: 10.1371/journal.pone.0301125 (PMC10977679; doi:10.1371/journal.pone.0301125)
Supplement: S1 Fig — Dotted line depicts mean screening rate. (DOCX) [file pone.0301125.s001.docx]

# Supplement File

Figure S1: 14-month documented mental health screening prevalence with corresponding 95% CI among different ages (patients aged >95 years were grouped into 95-year-olds). Dotted line depicts mean screening rate.
